# Transcriptome analysis insight into ethylene metabolism and pectinase activity of apricot (*Prunus armeniaca* L.) development and ripening

**DOI:** 10.1038/s41598-021-92832-6

**Published:** 2021-06-30

**Authors:** Min Xu, Weiquan Zhou, Wenjuan Geng, Shirong Zhao, Yan Pan, Guoquan Fan, Shikui Zhang, Yatong Wang, Kang Liao

**Affiliations:** 1 Research Centre of Characteristic Fruit Tree, College of Horticulture and Forestry, Xinjiang Agricultural University, Urumqi, 830052 Xinjiang China; 2 Xinjiang Academy of Agricultural Sciences, Urumqi, 830052 Xinjiang China; 3 Luntai National Fruit Germplasm Resources Garden of Xinjiang Academy of Agricultural Sciences, Luntai, 841600 Xinjiang China

**Keywords:** Plant sciences, Plant physiology, Transcriptomics

## Abstract

Ethylene metabolism is very important for climacteric fruit, and apricots are typical climacteric fruit. The activity of pectinase is closely related to fruit firmness, which further affects fruit quality. To better understand ethylene metabolism, pectinase activity and their molecular regulation mechanisms during the development and ripening of apricot fruit, ethylene metabolism, pectinase activity and the “Luntaibaixing” apricot fruit transcriptome were analyzed at different developmental stages. Ethylene metabolic precursors, enzyme activities and ethylene release increased during fruit development and ripening, with significant differences between the ripening stage and other stages (*P* < 0.05). Fruit firmness decreased significantly from the S1 to S5 stages, and polygalacturonase, pectin methylesterase, and pectin lyase activities were significantly higher in the S5 stage than in other stages. RNA sequencing (RNA-seq) analysis of fruit resulted in the identification of 22,337 unigenes and 6629 differentially expressed genes (DEGs) during development and ripening, of which 20,989 unigenes are annotated in public protein databases. In functional enrichment analysis, DEGs among the three stages were found to be involved in plant hormone signal transduction. Four key genes affecting ethylene metabolism, six key ethylene signal transduction genes and seven genes related to pectinase in apricot fruit were identified by KEGG pathway analysis. By RNA-sequencing, we not only clarified the molecular mechanism of ethylene metabolism during the ripening of "Luntaibaixing" apricot fruit but also provided a theoretical basis for understanding pectin metabolism in apricot fruit.

## Introduction

Apricot *(Prunus armeniaca* L*.*) fruit is rich in a variety of nutrients, including carotenoids, polyphenols, ascorbic acid and numerous trace elements^1^. Apricot has a high nutritional content, can be processed into a variety of food products and has become an important fruit worldwide^2^. Apricot fruit is a respiratory climacteric fruit^3^. Ethylene induces apricot fruit ripening, leading to a sharp decline in apricot fruit firmness and accelerated cell wall degradation in the softening process; thus, apricot fruit is not resistant to storage and transportation. Changes in ethylene metabolism in apricot fruit lead to serious cell wall degradation, resulting in fruit softening: the loss rate can reach 30–40%^4^.

In climacteric fruits, an ethylene peak indicates that the fruits are aging. Therefore, inhibiting or delaying the production of ethylene in apricot fruits is an important measure to prolong storage life^5^. Ethylene biosynthetic pathways have been well established in a number of previous studies. Methionine (MET) is transformed into S-adenosylmethionine (SAM) under the action of S-adenosylmethionine synthase, SAM is then converted into 1-aminocycloalanine-1-carboxylic acid (ACC) under the action of ACC synthase (ACS), and ACC is converted to ethylene by ACC oxidase (ACO)^6,7^. Among these pathway components, ACC content is a limiting factor for ethylene production, and ACS and ACO are rate-limiting enzymes in ethylene biosynthesis^8^. Therefore, it is of great significance to study the changes in ACC content, ACO activity and ACS activity during fruit development and maturity to understand ethylene biosynthesis in apricot fruit. After ethylene synthesis, fruit ripening is mediated by signal transduction.

The main ethylene signal transduction pathways are as follows: first, under the action of Cu^2+^, ethylene molecules bind to ethylene receptors (ethylene response 1 (ETR1), ethylene response sensor 1 (ERS1), ETR2, ERS2 and ethylene-insensitive 4 (EIN4)) located on the endoplasmic reticulum membrane, resulting in the activation of the negative regulatory component receptor constitutive triple response 1 (CTR1). After inactivation, the CTR1 receptor complex no longer phosphorylates the downstream signal component EIN2, but EIN2 is activated because it is not degraded. Then, the carboxyl end of the EIN2 protein (EIN2 CEND) is cleaved and dissociates, after which it enters the nucleus. EIN2 CEND may inhibit EIN3-binding F-Box 1/2 (EBF1/2) protein-mediated transcription factor ethylene-insensitive 3 (EIN3)/ethylene-in-sensitive-like 1 (EIL1)^9^. The ubiquitination degradation process promotes the accumulation of EIN3/EIL1 in the nucleus, and EIN3/EIL1 transcription then activates the expression of downstream target genes such as ethylene-responsive factor 1 (ERF1) to produce an ethylene response^10,11^.

Under the influence of ethylene, the firmness of the fruit will decrease, and the flesh will soften. Studies have shown that ethylene plays an important role in the ripening and softening of climacteric fruits^12^. Ethylene metabolism is closely related to fruit ripening and softening. During fruit ripening and softening, pectin in the cell wall of the fruit is generally gradually degraded, resulting in the destruction of the integrity of the cell wall structure and the softening of the fruit parenchyma^13^. The softening of fruits during storage is largely dependent on cell wall modification by various cell wall modification enzymes^14^. Cell wall-modifying enzymes, especially pectinase, are involved in the process of fruit ripening and softening; these enzymes include polygalacturonase (PG), pectin methylesterase (PME), pectin lyase (PL), cellulase (Cx) and galactosidase (β-galactosidase, β-Gal)^15^. These enzymes have been studied during the postharvest softening of many fruits, including strawberry^16^, blueberry^17^, grape^18^ and pear^19^. The available studies on ethylene metabolism, ripening and softening in apricot fruits and the related genes have mainly focused on postharvest fruit^20–22^, while research focusing on both ethylene metabolism and pectinase during apricot fruit development and ripening is rare. The firmness of apricot fruit gradually decreases during the ripening process. The reasons for the decrease in firmness can be summarized according to two aspects: the metabolism of ethylene, which is an important plant hormone inducing fruit ripening, and pectin degradation, which directly leads to fruit softening. The study of ethylene metabolism and pectinase activity is of great significance to ameliorate the loss and waste of apricot fruit during ripening. Exploring the key genes affecting these two aspects provides an important theoretical basis for ethylene regulation of apricot fruit ripening.

## Materials and methods

### Materials

*P. armeniaca* L (“Luntaibaixing”) apricot fruit was used as the experimental material. All experimental materials were obtained from the Luntai County fruit tree resource nursery, Xinjiang Academy of Agricultural Sciences in China. The apricot plants were grown with a row spacing of 3 m × 4 m in the east–west direction under conventional fertilizer and water management. From 14 days after full bloom, in the young fruit stage, to fruit ripening, fruits were collected from a total of 5 stages: the S1 stage (14 days after full bloom, young fruit stage, average temperature of 24 ℃), S2 stage (28 days after full bloom, expansion stage, average temperature of 29 ℃), S3 stage (42 days after full bloom, hard-core stage, average temperature of 33 ℃), S4 stage (63 days after full bloom, color changing stage, average temperature of 33 ℃) and S5 stage (77 days after full bloom, ripening stage, average temperature of 35 ℃). Fruits of the same size and appearance were selected from three sample plants in each stage. Some fruits were used to test fruit firmness and ethylene release, and some fruits were cut into small pieces and frozen in liquid nitrogen. The fruit samples were brought back to the laboratory and stored at − 80 °C.

### Fruit firmness

Ten fruits were randomly selected from each group, the equatorial epidermis of each fruit was cut off, and the flesh hardness (kg/cm^2^) of each fruit on the sunny side and the dark side was measured with a GY-1 (Lice, Jinan, China) fruit hardness tester. Three groups of replicates were included in the experiment.

### Measurement of ethylene production

According to the method of Guo et al.^23^ with slight modification, 0.5 kg of fruit was placed in a 2.25 L airtight container for 1 h, and 1 mL of headspace air was extracted. The results were determined by gas chromatography (GC) (Agilent 7890B GC system, Santa Clara, USA). Three biological replicates were performed for each sample.

The parameters of the gas chromatograph were as follows: in ethylene mode, column box temperature: 50.0 ℃, inlet (SS inlet) temperature: 200 ℃, pressure: 12.259 psi, flow rate: 18.00 mL/min, spacer purge flow rate: 3.0 mL/min. FID temperature: 300 ℃, flame: on, gas flow: 30.00 mL/min, practical gas flow: 40.00 mL/min, tail gas flow: 25.00 mL/min, signal value: 40.0 PA; rear detector temperature: 42.8 ℃, filament: off, practical gas flow rate: −0.026 mL/min, tail gas flow rate: 2.000 mL/min, signal value: 0.0 (25 μV); ethylene standard curve: y = 4.315x−0.1727, Y: ethylene peak area; X: ethylene concentration (μL/L).

### Measurement of ACC content, ACS activity and ACO activity

These measurements were performed with reference to the method of Hans Kende^24^. Fruit tissue was ground into a mortar with an appropriate amount of liquid nitrogen. A 0.1 g sample was weighed and extracted by adding 9 times the sample volume of 4 °C precooled phosphate buffer (pH 7.4). The supernatant was centrifuged at 4 °C and 8000 rpm for 30 min. The ACC content was then determined. Plant ACC, ACS and ACO ELISA kits (Ruishuo, Shanghai, China) were used to perform chromogenic reactions in strict accordance with the manufacturer’s instructions, and measurements were then carried out at 450 nm using a Rayto RT-6100 system (Rayto, Shenzhen, China). Three groups of biological replicates were performed for each sample.

### Measurement of polygalacturonase (PG) activity

A colorimetric method was used for PG activity determination^25^. First, a standard curve was generated, and the enzyme solution was then prepared. A 2.0 g fruit sample was weighed and placed in a precooled mortar, and 5 mL of precooled 95% ethanol was added. After grinding and homogenization in an ice bath, all of the samples were transferred to centrifuge tubes, subjected to low-temperature treatment at 4 °C for 10 min, and then centrifuged at 12,000 r/min for 20 min at 4 °C. The supernatant was poured off, and 5 mL of precooled 80% ethanol was added to the precipitate, followed by oscillation, low-temperature treatment for 10 min, and centrifugation under the same conditions indicated above. Thereafter, the supernatant was removed, another 5 mL of precooled extraction buffer was added to the precipitate, and the sample was placed at 4 °C for extraction for 20 min. The supernatant was collected after centrifugation as the enzyme extract and stored at 4 °C for later use. Then, 1.0 mL of 50 mmol/L pH 5.5 sodium acetate buffer and 0.5 mL of 1% polygalacturonic acid solution were added. The mixture was mixed and placed in a 37 °C water bath for 1 h. After heat preservation, 1.5 mL of 3,5-dinitrosalicylic acid reagent was quickly added, and the mixture was heated in a boiling water bath for 5 min. The mixture was then quickly cooled to room temperature, diluted with distilled water to 25 mL, and mixed well. The absorbance value at 540 nm was determined for the solution in each tube by colorimetry using the same method employed for the standard curve. Three replicates were performed for each sample.

### Measurement of pectin methylesterase (PME) activity and pectin lyase (PL) activity

Fruit tissue was ground and crushed in a mortar with an appropriate amount of liquid nitrogen, and 0.1 g of the sample was weighed. Then, the sample was extracted with 9 times the sample volume of 4 °C precooled phosphoric acid buffer (pH 7.4). The supernatant was centrifuged at 4 °C at 8000 rpm for 30 min, and the supernatant was collected as the enzyme extract. The enzyme activities of PME and PL were detected with ELISA kits (Ruishuo, Shanghai, China) in strict accordance with the operation steps specified in the kit. After the chromogenic reaction, enzyme activity was detected on a Rayto RT-6100 system (Rayto, Shenzhen, China) at 450 nm. Three groups of biological replicates were performed for each sample.

### RNA-seq analysis

#### RNA extraction

Total RNA was extracted from the apricot plant tissue using TRIZOL Reagent (St. Louis, Missouri, USA) according to the manufacturer’s instructions (Invitrogen, Waltham, USA), and genomic DNA was removed using DNase I (TaKaRa, Dalian, China). Then, RNA quality was determined by a 2100 Bioanalyzer (Agilent, Santa Clara, USA) and quantified using an ND-2000 (NanoDrop 2000, Wilmington, DE, USA). Only a high-quality RNA sample (OD260/280 = 1.8–2.2) was used to construct a sequencing library.

#### Library preparation and sequencing

The RNA-seq transcriptome library was prepared following the TruSeqTM RNA sample preparation kit (Illumina, San Diego, CA) using 1 μg of total RNA. Briefly, messenger RNA was first isolated by oligo(dT) beads according to the polyA selection method and then fragmented by fragmentation buffer. Second, double-stranded cDNA was synthesized using a SuperScript double-stranded cDNA synthesis kit (Invitrogen, Waltham, USA) with random hexamer primers (Illumina, San Diego, CA). Then, the synthesized cDNA was subjected to end repair, phosphorylation and ‘A’ base addition according to Illumina’s library construction protocol. Libraries were size-selected for cDNA target fragments of 200–300 bp on 2% Low Range Ultra Agarose followed by PCR amplification using Phusion DNA polymerase for 15 PCR cycles. After quantification by TBS380, the paired-end RNA-seq sequencing library was sequenced with an Illumina NovaSeq 6000 sequencer (San Diego, California, USA).

#### Read mapping

The raw paired-end reads were trimmed and quality controlled by SeqPrep (https://github.com/jstjohn/SeqPrep) and Sickle (https://github.com/najoshi/sickle) with default parameters. Then, clean reads were separately aligned to the reference genome with orientation mode using TopHat (http://tophat.cbcb.umd.edu/, version 2.0.0)^26^ software. The mapping criteria of bowtie were as follows: sequencing reads should be uniquely matched to the genome, allowing up to 2 mismatches without insertions or deletions. Then, the gene region was expanded according to the depths of the sites, and the operon was obtained. In addition, the whole genome was split into multiple 15 kb windows that shared 5 kb. New transcribed regions were defined as more than 2 consecutive windows without overlapping regions of genes, where at least 2 reads were mapped per window in the same orientation.

#### Differential expression analysis and functional enrichment

To identify DEGs (differentially expressed genes) between two different samples, the expression level of each transcript was calculated according to the fragments per kilobase of exon per million mapped reads (FRKM) method. RSEM (http://deweylab.biostat.wisc.edu/rsem/)^27^ was used to quantify gene abundances. The R statistical package software EdgeR (Empirical analysis of Digital Gene Expression in R, (http://www.bioconductor.org/packages/2.12/bioc/html/edgeR.html)^28^ was utilized for differential expression analysis. In addition, functional enrichment analysis, including GO and KEGG analyses^29^, was performed to identify which DEGs were significantly enriched in GO terms and metabolic pathways at a Bonferroni-corrected *P*-value ≤ 0.05 compared with the whole-transcriptome background. GO functional enrichment and KEGG pathway analysis were carried out by Goatools (https://github.com/tanghaibao/Goatools) and KOBAS (http://kobas.cbi.pku.edu.cn/home.do)^30^.

All RNA-seq data were deposited in the NCBI SRA database under accession number PRJNA723373.

### qRT-PCR analysis

Real-time quantitative PCR (qRT-PCR) analysis was conducted with apricot fruit RNA extracted according to standard RNA extraction steps. All primers used in qRT-PCR analysis were synthesized by China Majorbio Co., Ltd. (Table S1). A CFX Connect™ Real-time PCR detection system (Bio-Rad, Beijing, China) was used. The analysis of each sample was repeated three times, and the 2^−ΔΔCT^ method was used for quantitative data analysis^31^.

### Data statistics and analysis

All data are expressed as the means ± standard deviations of three replicates. The ethylene release, ethylene metabolism, fruit firmness and pectin metabolism data were analyzed by one-way ANOVA (SPSS Inc., Chicago, IL, USA). Duncan’s test with a significance level of 0.05 was used. Microsoft Excel 2010 was used to calculate the standard error (SES), and Microsoft Excel 2010 and Origin 8.0 software were used to produce charts.

## Results

### Ethylene synthesis and metabolism at different growth stages of fruit

**Figure 1:**
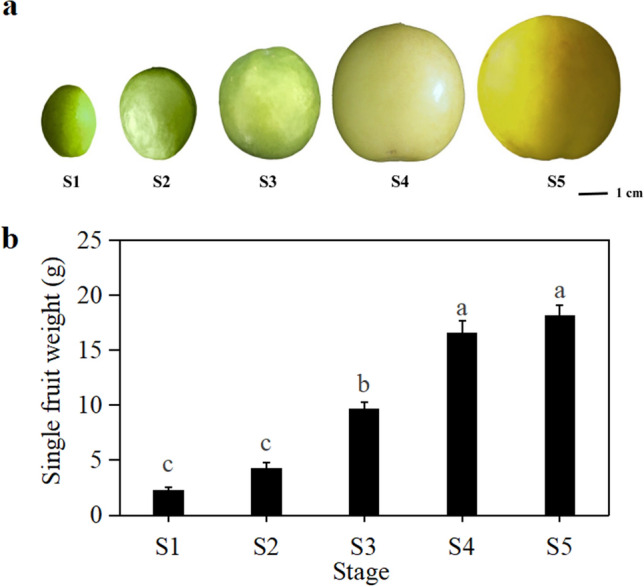
Changes in the fruits and weight of "Luntaibaixing" at different developmental stages (a) Fruit development in 5 stages; (b) Single fruit weight. Note: different letters indicate significant differences at *P* < 0.05.

**Figure 2:**
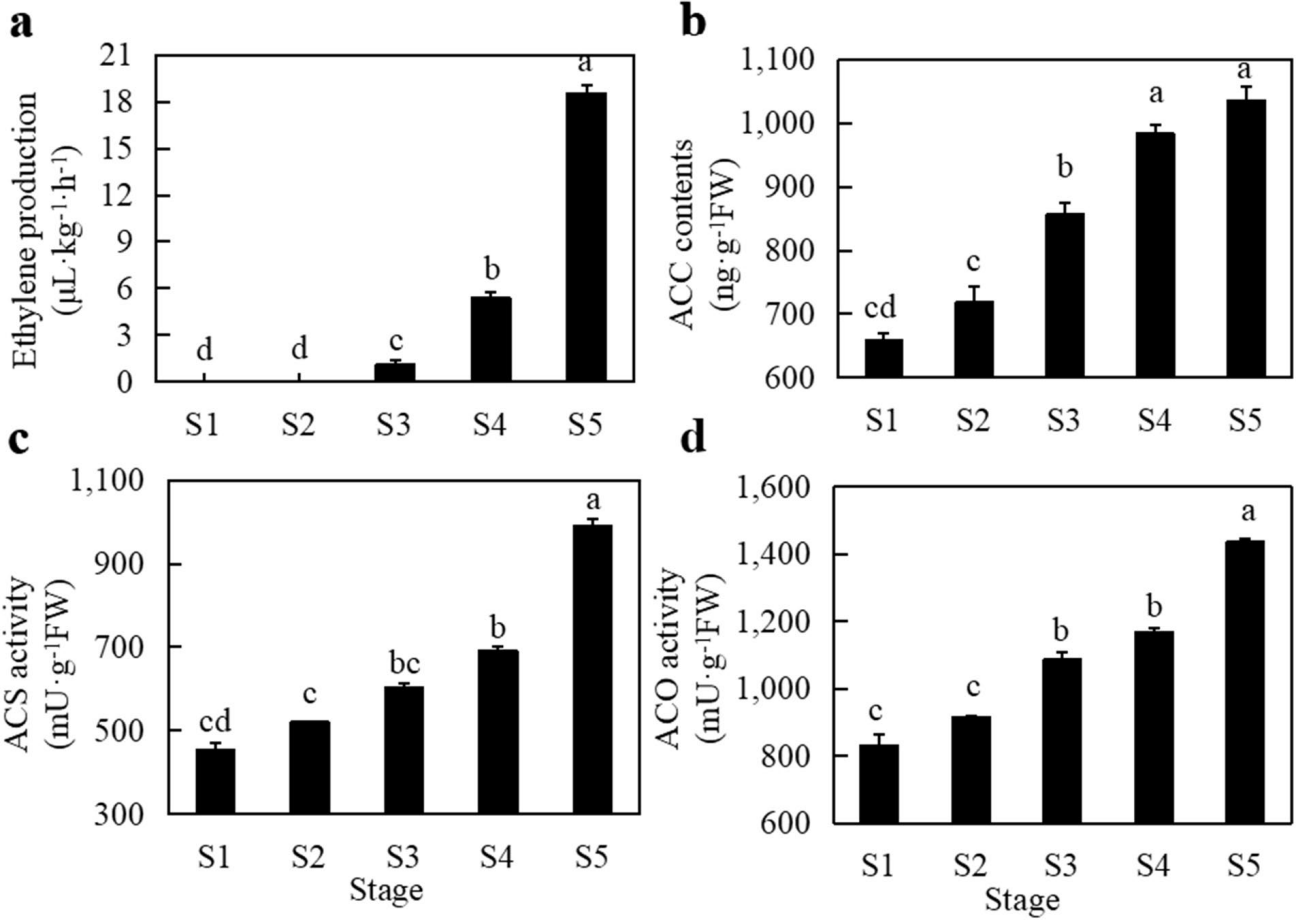
Changes in the fruits and ethylene metabolism of "Luntaibaixing" at different developmental stages (a) Ethylene release; (b) ACC content; (c) ACS activity; (d) ACO activity. Different letters indicate significant differences at *P* < 0.05.

Apricot fruit from the “Luntaibaixing” cultivar at five developmental and ripening stages (S1; S2; S3; S4; S5) were subjected to analysis of ethylene metabolism and pectinase activity (Fig. 1a). The single fruit weight showed a significant difference in S1–S5 (*P* < 0.05) (Fig. 1b). Ethylene release increased from S1 to S5 with fruit development (Fig. 2a). Ethylene release in the first two stages (S1 and S2) of fruit development was undetectable. There was no significant difference in ethylene release between the two stages. There was little ethylene production in the S3 stage, which highlighted a significant difference from the previous two stages (*P* < 0.05). In the last two stages (S4 and S5), ethylene release increased by 79.64% and 94.01%, respectively. Ethylene release peaked at S5 and reached 18.492 μL·kg^−1^·h^−1^, which was significantly different from the release measured in other stages (*P* < 0.05). The S5 stage is the fruit maturity stage. The content of ACC, the precursor for ethylene synthesis, showed a trend of gradual increase with the development of fruits (Fig. 2b). At S5, the content of ACC reached the maximum value of 1035.807 ng·g^−1^ FW, which was significantly different from the values in S1–S3 (*P* < 0.05). The content of ACC in S1 and S2, the first two stages of fruit development, was low. The content of ACC in the S3 stage increased significantly and was 1.2 times that in the S2 stage. The ACC contents of S4 were significantly different from those of S1 to S3 (*P* < 0.05). The activity of ACS, a key enzyme in ethylene synthesis, also showed an increasing trend with fruit development (Fig. 2c). ACS activity increased during the S1-S3 stages of fruit development, but the difference was not significant. ACS activity was significantly increased during the turning stage (S4) and reached a maximum value of 990.832 mU·g^−1^ FW at S5, which was significantly different from the values in the other four stages (*P* < 0.05). The activity of ACO, the rate-limiting enzyme in ethylene synthesis, showed a gradual increasing trend from S1 to S5 (Fig. 2d). The increasing trend of ACO activity in fruit could be divided into three stages: S1-S2 belonged to the first stage, when ACO activity in fruit was low, and the difference was not significant; S3-S4 belonged to the second stage, when ACO activity was increased significantly as compared with the first two stages (*P* < 0.05); and S5 belonged to the third stage, when fruit ACO activity reached its maximum level of 1438.256 mU·g^−1^ FW, which was significantly different from the activity in the other four stages (*P* < 0.05).

### Changes in fruit firmness and pectinase activity during different growth stages

**Figure 3:**
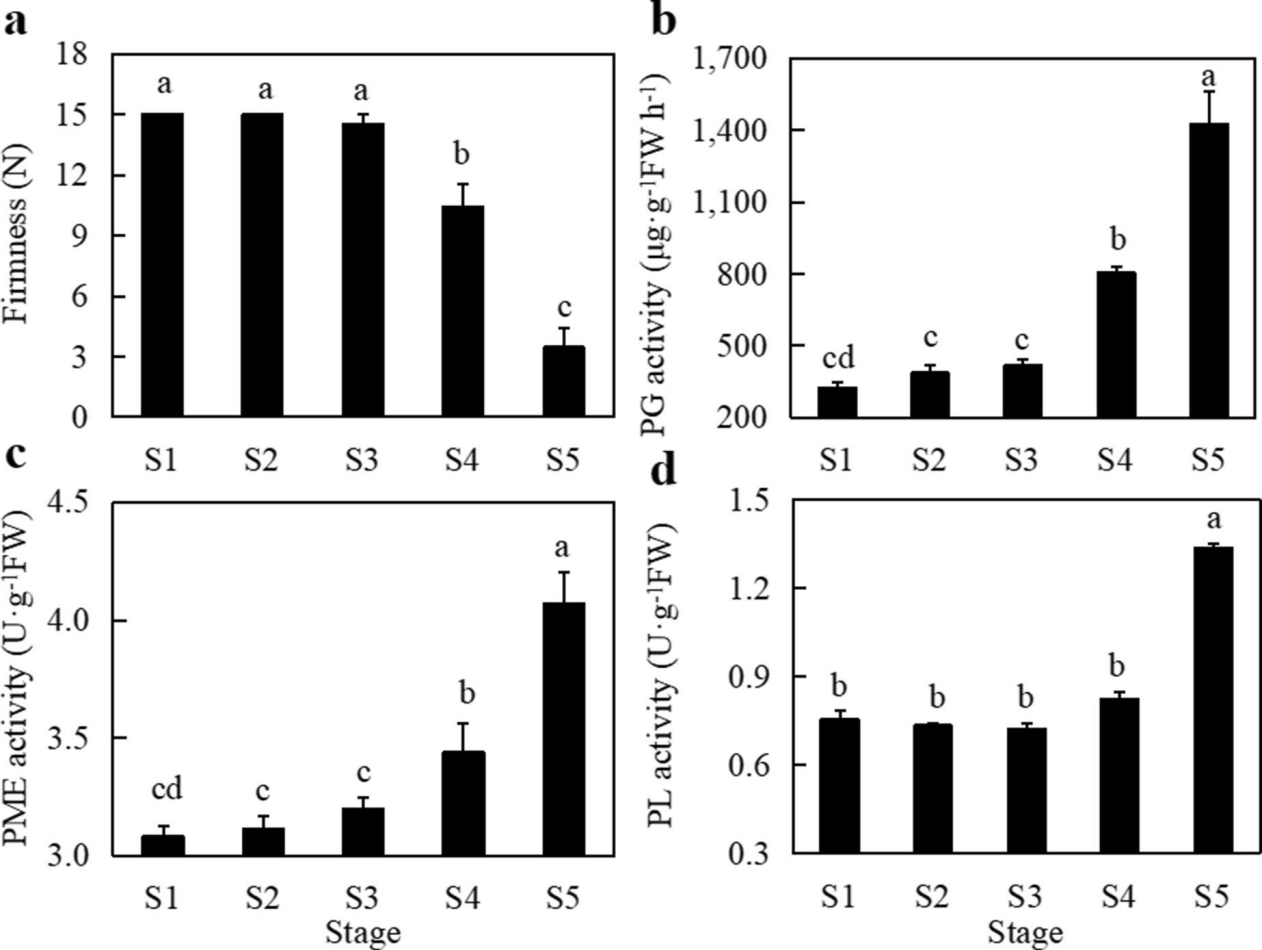
Numbers of RNA-seq transcriptome reads in different length intervals.Changes in fruit firmness in different developmental and mature stages (a); polygalacturonase activity; PG activity (b); pectin methyl esterase activity; PME activity (c); pectin lyase activity; PL activity (d). Different letters indicate significant differences at *P* < 0.05.

Fruit firmness showed a decreasing trend from S1 to S5. The lowest firmness was 3.47 kg/cm^2^ in S5, which was significantly lower than the firmness in S1-S4 (*P* < 0.05) (Fig. 3a). During the first three stages of fruit development, fruit firmness did not change significantly. At S4, fruit firmness decreased to a certain extent and was significantly lower than that in the first three periods. With the further ripening of fruit, the firmness of fruit in the S5 stage decreased by 66.73%. A decrease in fruit firmness is one of the indicators of fruit ripening and is closely related to the activity of pectinase metabolism. The main pectin-metabolizing enzymes affecting fruit firmness include PG, PME and PL. PG enzyme activity increased from S1 to S5 with the development and ripening of fruit and reached a maximum at S5, which was significantly different from the activity measured in S1 to S4 (Fig. 3b). PG activity in S1-S3 was very low in the early stage of fruit development, and there was no significant difference among the three stages (*P* > 0.05). The activity of PG in S4 and S5 was significantly higher than during the other three periods. The change trends of PME enzyme activity and PG enzyme activity were consistent, showing increasing trends reaching a peak during the S5 stage (Fig. 3c). The PME activity in S1-S3, the early stage of fruit development, was very low, and there was no significant difference among these three stages (*P* > 0.05). PME activity increased significantly during S4–S5. The change in PL activity was different from those of the other two enzymes during fruit development. There was no significant difference in PL activity among S1–S4, but PL activity in S5 was significantly higher than that in the other four stages (Fig. 3d). The activities of the three pectin-metabolizing enzymes in the fruit differed, but all three pectin-metabolizing enzymes showed significantly increased activities in the S5 stage compared with the other stages, resulting in a significant decrease in fruit firmness in the S5 stage.

### Transcriptome sequencing analysis

#### Quality assessment of sequencing data

Using the Illumina NovaSeq 6000 platform, the raw data obtained from 9 samples were filtered, and a total of 4.3–6.9 million clean reads were obtained (Table S2). A total of 67.58 GB of clean sequencing data were obtained, with more than 6.37 GB of clean data per sample, and the Q30 base percentage was more than 93.49%. The Q30 base percentage was greater than 93%, and the percentage of G and C bases among the total bases was 45.85–46.29% (Table 1). The reference gene source was *Prunus_armeniaca,* and the reference genome version was parmeniaca_v1.0^2^. The clean reads of each sample were compared with the designated reference genome, and the alignment rate ranged from 89.53 to 92.95%. The data showed that the RNA-seq results were of high quality overall and could be used for further analysis.

**Table 1:** Statistics on the quality and output of the RNA-Seq libraries.

Sample	Clean reads	Clean bases	Error rate (%)	Q20 (%)	Q30 (%)	GC content (%)
L_42D_1	44305872	6544907152	0.0252	97.99	93.9	45.85
L_42D_2	43371186	6367763328	0.025	98.07	94.07	45.86
L_42D_3	50223490	7398236242	0.0252	97.99	93.89	46.01
L_63D_1	45857002	6781731099	0.0254	97.9	93.74	46.04
L_63D_2	48242612	7116891165	0.0252	97.97	93.86	45.99
L_63D_3	52068768	7723835674	0.0253	97.98	93.83	46.22
L_77D_1	50750106	7546923514	0.0255	97.88	93.57	46.19
L_77D_2	53070390	7899235443	0.0256	97.84	93.49	46.29
L_77D_3	69605330	10198744101	0.025	98.08	94.08	46.21

The genes identified through transcriptome sequencing in the nine fruit samples of “Luntaibaixing” were annotated in NCBI's non-redundant (Nr) protein sequence database, Gene Ontology (GO), Clusters of Orthologous Groups of proteins (COG), and Kyoto Encyclopedia of Genes and Genomes (KEGG), and the numbers and proportions of genes differed among the databases (Table 2). The greatest number of annotated genes was obtained in the NR database, in which 21,891 genes were annotated, accounting for 98.00% of the total genes. The fewest annotated genes were obtained in the KEGG database, in which 8491 genes were annotated, accounting for 38.01% of the total genes. The numbers and percentages of genes annotated in the other four databases were similar.

**Table 2:** Success rates of gene annotation in different databases.

	Express gene number (%)	Express transcript number (%)	All gene number (%)	All transcript number (%)
GO	16651 (0.7454)	42731 (0.7819)	21658 (0.6812)	51862 (0.7485)
KEGG	8491 (0.3801)	23670 (0.4331)	10171 (0.3199)	27750 (0.4005)
COG	15608 (0.6988)	40854 (0.7476)	18395 (0.5786)	47464 (0.685)
NR	21891 (0.98)	53860 (0.9856)	30525 (0.9601)	67600 (0.9756)
Swiss-Prot	17448 (0.7811)	44259 (0.8099)	21393 (0.6729)	52500 (0.7577)
Pfam	17935 (0.8029)	44888 (0.8214)	22577 (0.7101)	53769 (0.776)
Total_anno	21899 (0.9804)	53871 (0.9858)	30547 (0.9608)	67626 (0.976)
Total	22337 (1.0)	54649 (1.0)	31792 (1)	69289 (1)

#### Analysis of DEGs in three fruit stages

**Figure 4:**
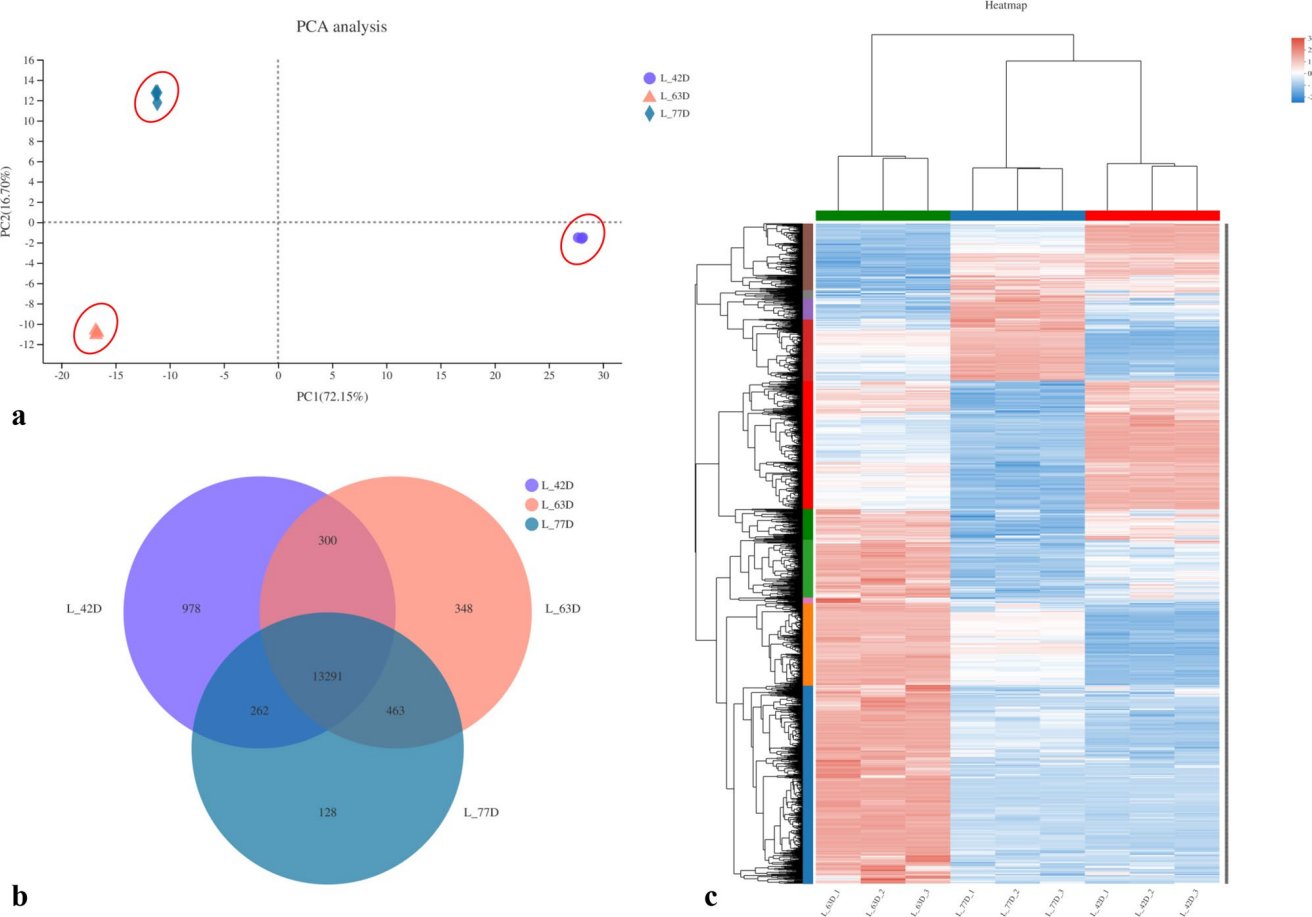
PCA diagram, Venn diagram (**b**) and heat map of the correlation (**c**) of DEGs among 3 stages of “Luntaibaixing” fruit.

Principal component analysis (PCA) can identify outlier samples and evaluate the repeatability of the samples. The 9 samples examined in our study were divided into three categories consistent with the three developmental stages of fruits (Fig. 4a). The number of common genes in the three stages and the number of unique genes in each period are shown in a Venn diagram of the samples (Fig. 4b). There were 978 unique genes expressed at S3 (L42D), 348 unique genes expressed at S4 (L63D), and 128 unique genes expressed at S5 (L77D). A total of 13,291 genes were co-expressed during all three stages. The expression levels of the DEGs identified between L42D (S3), L63D (S4) and L77D (S5) were plotted in the heat map (Fig. 4c).

#### KEGG and GO analyses of DEGs in the three stages

**Figure 5:**
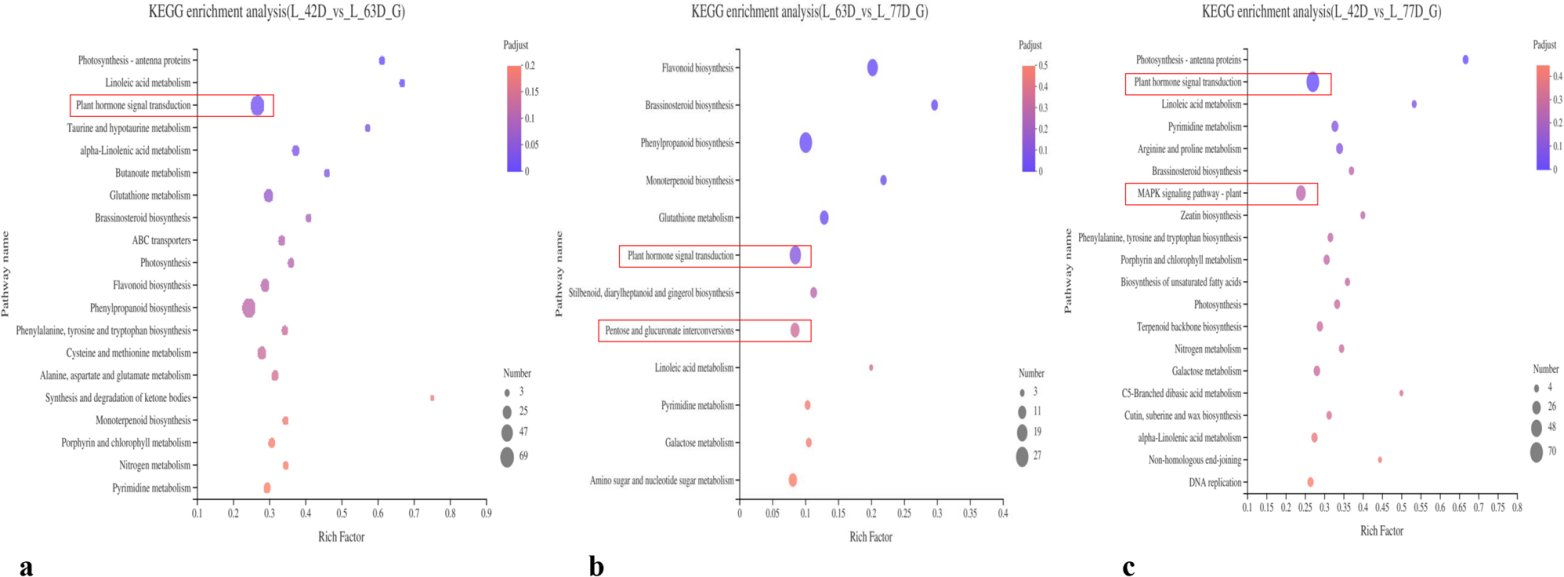
KEGG enrichment of annotated DEGs among 3 stages of “Luntaibaixing” fruit.

KEGG pathway enrichment analysis was conducted for the fruit DEGs identified between the three stages (Fig. 5a–c). The KEGG pathway analysis showed that 12–20 metabolic pathways were enriched among the DEGs identified in L42D vs. L63D, L63D vs. L77D and L42D vs. L77D. Among these pathways, the main metabolic pathway in which the DEGs were enriched according to paired comparisons of the three stages was plant hormone signal transduction; the numbers of genes enriched in this pathway were as follows: L42D vs. L63D (69 genes), L63D vs. L77D (22 genes), and L42D vs. L77D (70 genes). The main metabolic pathway related to the L63D vs. L77D DEGs was pentose and glucuronate interconversion, in which 14 DEGs were enriched (Fig. 5b). The main enriched metabolic pathway of the L42D vs. L77D DEGs was the plant MAPK signaling pathway, which included 39 DEGs (Fig. 5c). The pathways enriched among the DEGs identified between two adjacent stages among the three stages were all related to the metabolism and signal transduction of plant hormones.

**Figure 6:**
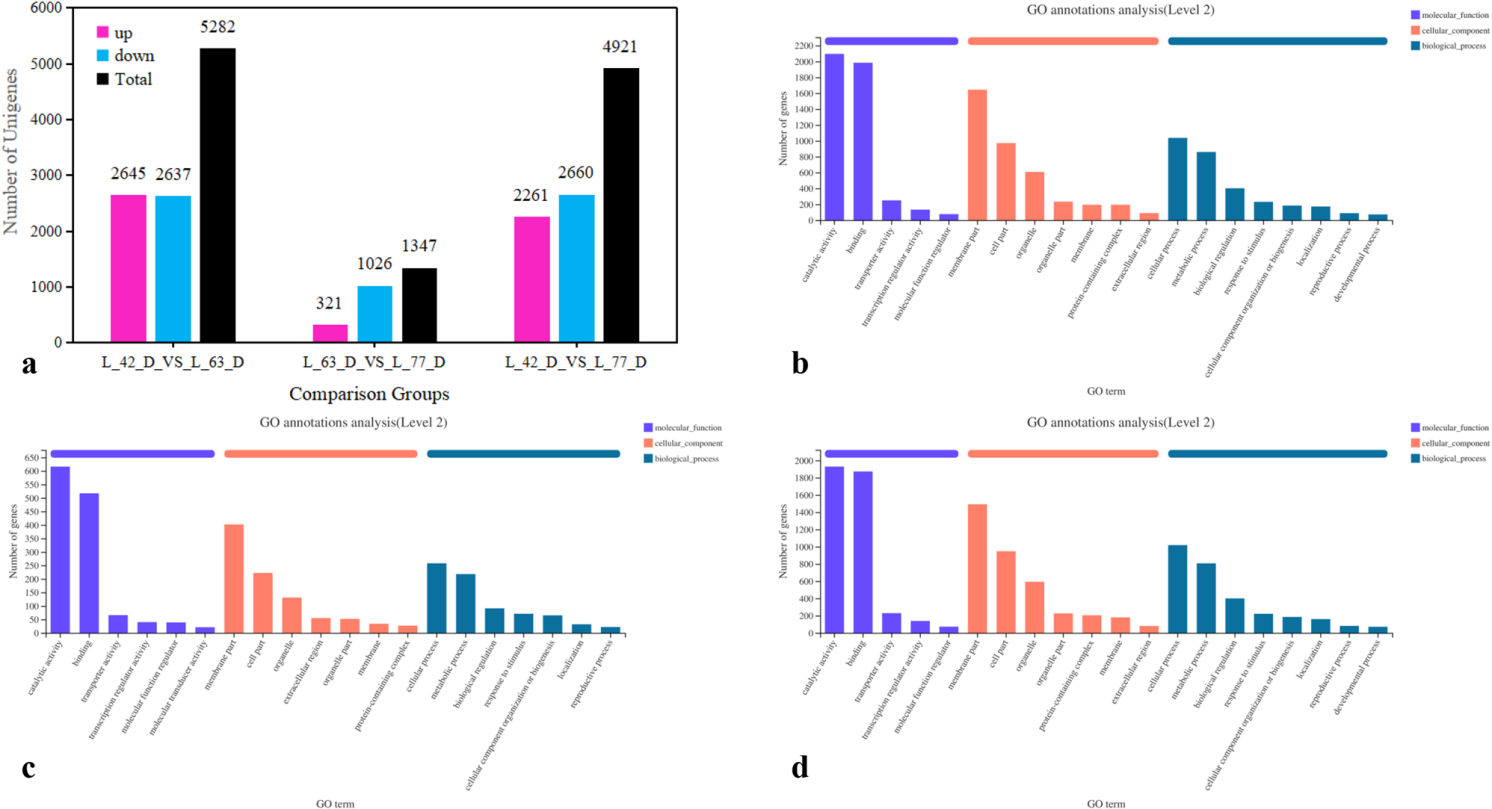
Numbers of up- and down-regulated DEGs and their functional annotation among 3 stages of “Luntaibaixing” fruit.

In the comparison of L42D and L63D, the number of upregulated genes was 2645, and the number of downregulated genes was 2637, but the difference in the former was more significant than that in the latter (Fig. 6a). In the comparison of L63D and L77D, the number of upregulated genes (321) was lower than that of downregulated genes (1026), and the difference in upregulated genes was significantly smaller than that in downregulated genes (Fig. 6a). The comparison of DEGs in L42D and L77D showed that the number of upregulated genes (2261) was lower than the number of downregulated genes (2660), but in terms of significance, the difference in the upregulated genes was more significant than that in the downregulated genes (Fig. 6a).

According to GO classification, the DEGs were divided into three functional categories: molecular function, biological process and cellular component (Fig. 6b–d). Among the three functional categories, 2790 genes (L42D vs. L63D) and 1,570 genes (L63D vs. L77D) were clustered in catalytic activity, and 2712 genes (L42D vs. L63D) and 1476 genes (L63D vs. L77D) were clustered in binding, after which the categories associated with the most genes were membrane part, cell part, organelle, metabolic process, etc.

### Analysis of ethylene metabolism and the pectinase metabolism pathway

**Figure 7:**
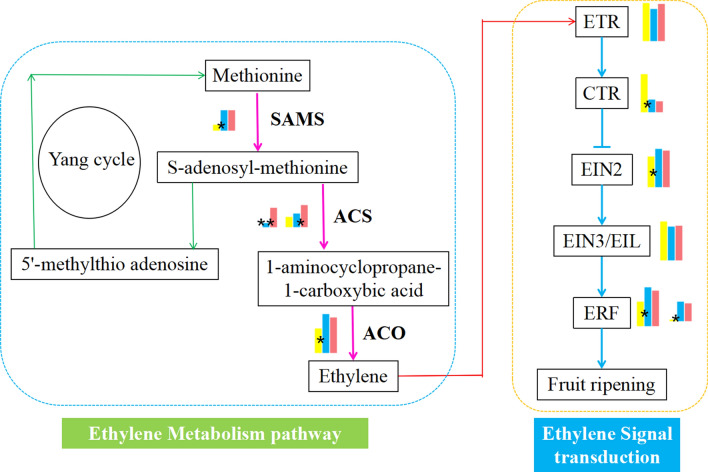
Ethylene metabolism and ethylene signal transduction pathways. Note: Green right arrow Yang cycle, Pink right arrow Ethylene metabolism, Blue right arrow Ethylene signal transduction, blue lined vertical negative regulation, blue dotted line Ethylene metabolism, yellow dotted line Ethylene signal transduction, yellow square the FPKM of S3, blue square the FPKM of S4, pink square the FPKM of S5.

Ten genes related to ethylene metabolism and ethylene signal transduction were identified by comparing the DEGs of apricot fruits in 3 developmental stages with six public databases (KEGG, NR, SwissProt, KOG, GO and Pfam), and an ethylene metabolism and signal transduction pathway map of apricot fruits was constructed. Each gene showed different expression levels in the 3 stages (Fig. 7). Four genes were associated with ethylene metabolism. These genes included one SAM synthetase gene (SAMS), and the expression levels of SAMS in L42D and L63D were significantly different. Additionally, there were two ACS (PARG27229, PARG18370) genes (Table S3), and their expression levels were significantly different in the three stages. There was one ACO (PARG13808) gene (Table S3), which showed a significant difference in expression between L42D and L63D. Six genes related to ethylene signal transduction were identified. One ETR gene was included in this group, which showed no significant difference in expression among the three periods. There was one CTR gene, and the expression of this gene was significantly different among L42D, L63D and L77D. There was one EIN2 gene, and the expression level of this gene was significantly different between L42D and L63D. There was one EIN3/EIL gene, which showed no significant difference in expression among the three stages. There were two ERF genes that showed significant differences in expression in L42D, L63D and L77D.

**Figure 8:**
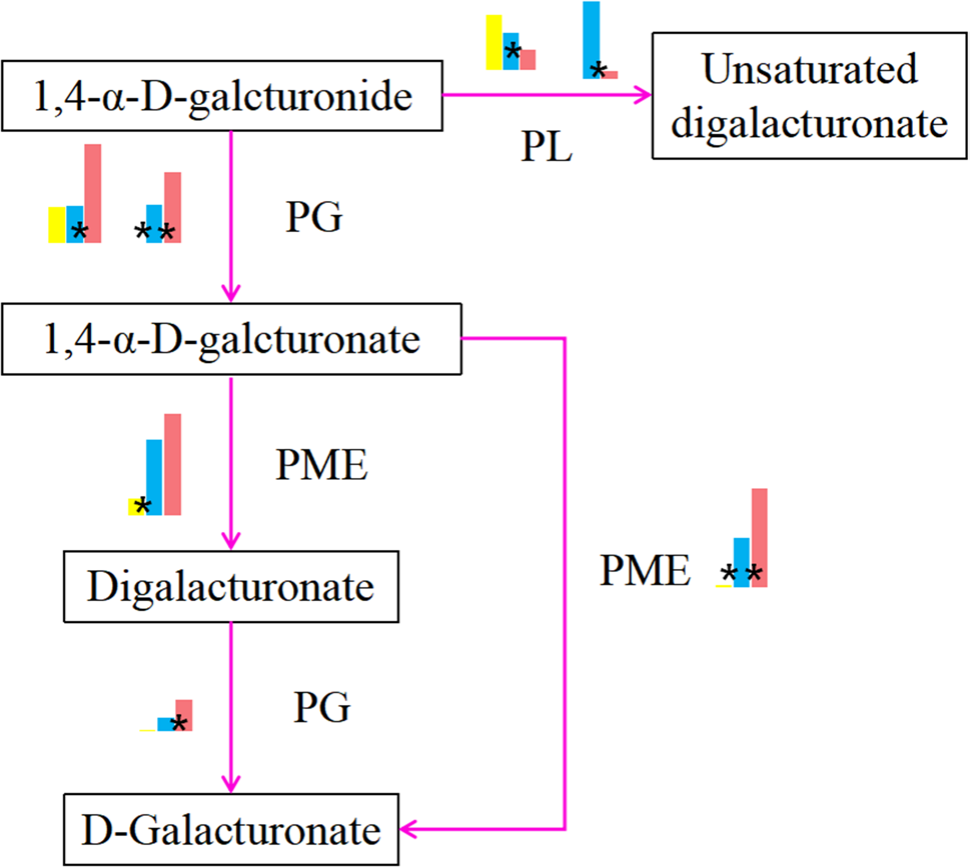
Pectinase metabolism pathway. Note: pink right arrow Pectinase metabolism, yellow square the FPKM of S3, blue square the FPKM of S4, pink square the FPKM of S5.

Seven genes in the pectin metabolism pathway were identified (Fig. 8). Among these genes were three PG enzyme (PARG12928, PARG10145 and PARG20309) genes (Table S3), two PME enzyme genes and two PL enzyme genes. Two of the three PG genes played a role in the metabolism of 1,4-α-D-galacturonate, produced from 1,4-α-D-polygalacturonide under the action of PG, and their expression levels were significantly upregulated in L42D and L63D and were insignificantly higher in L77D. One PG gene played a role in the transformation of digalacturonate into D-galacturonate. The expression level of the PG gene was significantly upregulated in L42D and L63D, but the expression level remained relatively low. One of the two PME genes played an important role in the transformation of 1,4-α-D-galacturonate into digalacturonate. Its gene expression was high in the three stages, and the gene was significantly upregulated in L42D and L63D. Another PME (PARG27582 and PARG19839) gene (Table S3) played an important role in the transformation of 1,4-α-D-galacturonate into D-galacturonate. The expression of the PME gene was significantly different in the three stages, showing high expression in L63D and L77D. 1,4-α-D-galacturonate produces unsaturated digalacturonate under the action of the PL enzyme. Two PL enzyme genes were involved in this process, and the DEGs were significantly downregulated.

### Validation of genes related to ethylene and pectinase metabolism by qRT-PCR

**Figure 9:**
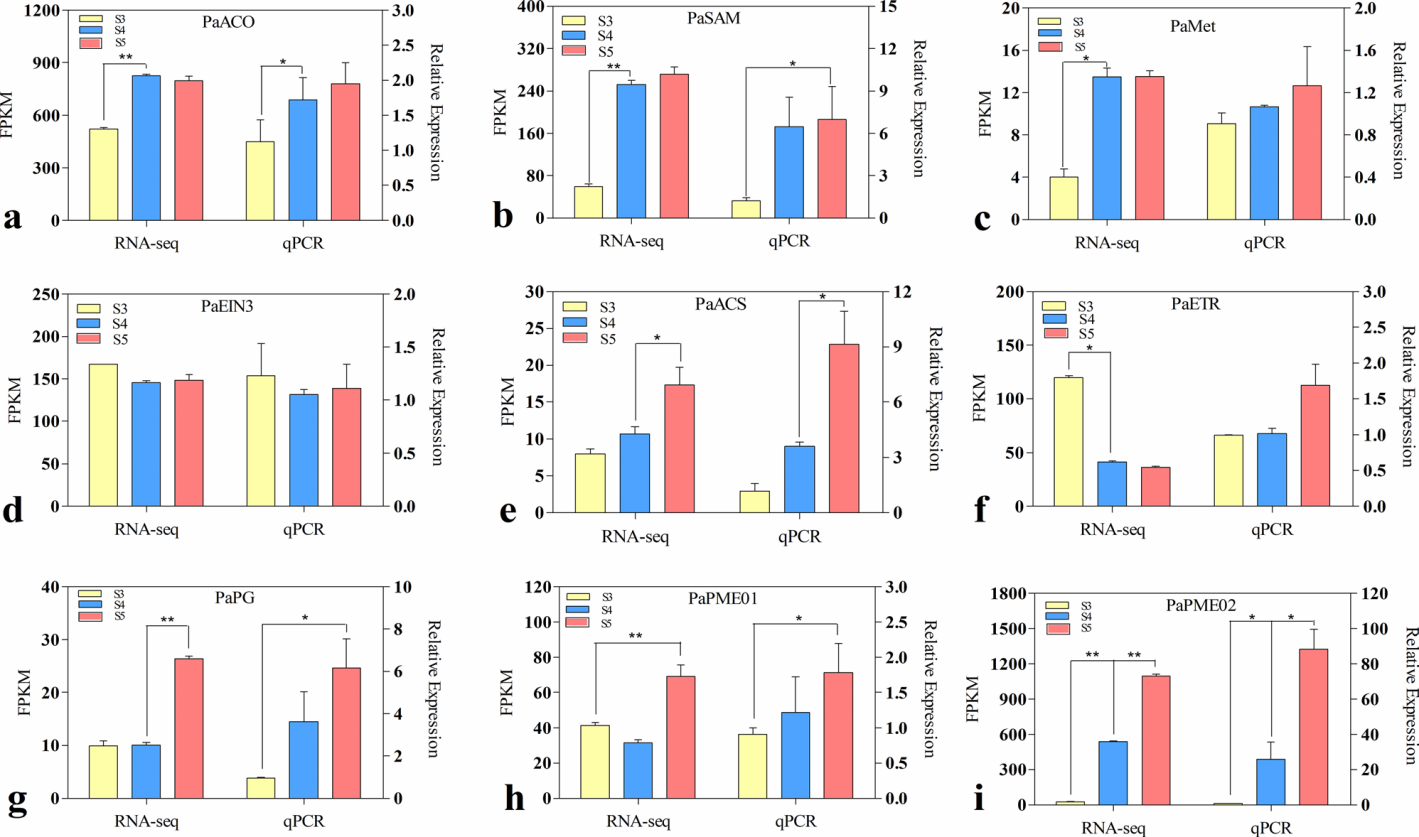
Verification of ethylene and cell wall metabolism-related DEGs by qRT-PCR. Note: All data are presented as means of three biological replicates, and error bars represent ± standard deviation. * indicates that there were significant differences between the two apricot cultivars during the same period (Student's unpaired *T* test; *P* < 0.05), and no * indicates that there were no significant differences.

To evaluate the validity of the RNA-seq data and further confirm the identified differential gene expression patterns, nine genes with expression differences in the three periods were selected for qRT-PCR analysis (Fig. 9). The transcription levels of PaACO, PaSAM, PaMet, PaACS, PaPG, PaPME01, and PaPME02 were lower in L42D in "Luntaibaixing" apricot, which was consistent with the downregulation trend observed via qRT-PCR. Their expression increased significantly in L63D or L77D, which reflected the changes in ethylene production and pectin degradation. Therefore, the qRT-PCR results were consistent with the RNA-seq results.

## Discussion

Ethylene plays important roles in ripening and quality development in climacteric fruits and serves key roles in fruit ripening and the physiological and biochemical changes that occur during storage^32^. In previous studies on ethylene biosynthesis and metabolism in climacteric fruits, it was observed that ethylene metabolism involves two systems: system I is responsible for the production of trace ethylene, and system II is responsible for the production of large amounts of ethylene mediated by the self-catalysis of trace ethylene^33^. Ethylene release in "Luntaibaixing" apricot fruit was studied in five developmental stages by GC. The results showed that in S4, the color change stage, system I produced a small amount of ethylene, and in S5, the ripening stage, system II synthesized a large amount of ethylene mediated by the self-catalysis of a small amount of endogenous ethylene, which accelerated the ripening and senescence of apricot fruit. These results are consistent with previous studies on ethylene metabolism in climacteric fruits^34^. In our study, ACS and ACO were found to be the key and rate-limiting enzymes of ethylene metabolism in "Luntaibaixing" apricot. The activities of ACS and ACO showed an upward trend from the young fruit stage to the ripening stage (S1-S5). The activities of ACS and ACO during the ripening stage and the ethylene release rate during the ripening stage showed the same trend, indicating that the two enzymes played important roles in ethylene biosynthesis in apricot fruit. This inference is consistent with the conclusion of Yang^33^ and Kan^35^ regarding the changes in ACS and ACO activity observed in the study of ethylene synthase and oxidase in climacteric fruits. Fruit texture softening is due to a change in the content of pectin, which is the main component of the cell wall. Polygalacturonase, pectin lyase and pectin methylesterase are involved in the changes in pectin content in fruit^36^. PG is a synthetic enzyme involved throughout the whole process of fruit ripening, and the PG gene is a specific expression product of the physiological stage of fruit ripening. PG activity is closely related to fruit firmness in different fruit varieties or different fruit ripening stages of the same variety, and PG is an important enzyme contributing to fruit softening^37^. Significantly negative correlations were found between fruit firmness and PG enzyme activity in “Luntaibaixing” apricot. Correlation analysis indicated that the PG enzyme played an important role in apricot fruit texture softening during ripening. This is consistent with Zhou's research on the relationship between fruit texture softening and pectinase during ripening^38^. PME plays a decisive role in determining the pectin content of fruit^39^. The PME-catalyzed methylation of pectin is necessary for PG activity. Therefore, the role of PME is mainly to prepare hydrolytic substrates for PG^40^, so the change in PME activity occurs earlier than that in PG. Some studies have indicated that PME activity shows a sharp upward trend in the early fruit ripening stage of Prunus, such as in peaches and cherries^14,41^, which is consistent with the conclusion that PME activity in "Luntaibaixing" apricot fruit increases significantly during the color change stage (S4). PL is involved in fruit ripening and softening by degrading demethylated pectin in the cell wall. Three kinds of pectin-metabolizing enzymes interact with each other to modulate pectin content. They are closely related to changes in fruit texture and softening during fruit ripening. Fruit ripening is an extremely complex process regulated by the genetic mechanisms of individual species. Fruit ripening is internally driven by endogenous hormones according to the developmental stages of fruits and is also affected by environmental stimulation^42^. The plant hormone ethylene plays an important role in the ripening and quality formation of climacteric fruits^43^. Ethylene can initiate and promote the generation of multiple metabolic substances and physiological and biochemical changes, including changes in color, texture, flavor, aroma, etc. Many transcription factors related to ethylene sensing, ethylene signal transduction and ripening promotion have been identified in tomato fruit and Arabidopsis^44^. Due to the highly conserved sequences of Rosaceae plants, previous studies on the transcriptome sequencing of apricot fruits at different stages have used peach or plum as the reference genome and have achieved gene alignment rates of transcriptome sequencing of 70% or more^45^. The reference genome used in this study was the apricot fruit genome^46^, and the gene alignment rate of the transcriptome was 87% or more, indicating an improvement of not only the gene alignment rate, but also the quality of apricot fruits. The precise study of apricot-specific genes is very helpful. In a study on the ethylene synthesis process by P. Muñoz and Wang, PaACS2 was shown to be the key gene in ethylene synthesis^47,48^. Our results showed that the expression levels of PaACS1 and PaACS2 in the ACS gene family related to ethylene biosynthesis were significantly upregulated; these genes exhibited especially significant differences in expression between S3 and S4 in the hard-core stage, which indicated that the physiological and biochemical changes occurring from S3 to S4 in the hard-core stage of apricot fruit were basically in accordance with the conditions of the ripening stage. Grimplet^49^ found that ACC oxidase (PA9TC1) was upregulated during apricot fruit ripening on the basis of 13,006 transcriptome markers, and the upregulation of the expression of this gene was increased before ethylene production, which was consistent with the conclusion that the PaACO gene was upregulated during fruit ripening in "Luntaibaixing" apricot (Table S3).

In the ethylene signal transduction pathway, the ethylene receptor ETR is located in the endoplasmic reticulum and transmits ethylene signals to the downstream element CTR1^50^. The expression level of ETR was shown to decrease slightly, but not significantly, which was slightly different from the expression of ETR observed by Zhang et al.^51^. A loss-of-function mutation of CTR1 leads to a constitutive ethylene reaction, which indicates that CTR1 is a negative regulator, and the phosphorylation of the substrate by CTR1 is obviously necessary to inhibit the ethylene reaction^52,53^. In our study, the expression of the CTR (PARG08711) gene showed a significant downward trend which was related to the negative regulation of CTR in the process of signal transduction. Zhang^54^ identified 15 transcription factors and ripening-related factors among apricot fruit genes via transcriptome sequencing of different developmental stages of “Jianali” apricot fruit, including the ethylene response factor ERF. Primary and secondary metabolism in fruit is regulated by AP2/ERF^55^. ERF1 plays an important role in the process of ethylene signal transduction. ERF1 can activate the downstream ethylene response, which is consistent with the conclusion that Pa ERF2 (PARG03663), a key gene in ethylene signal transduction, can activate the response of fruit to ethylene and lead to fruit ripening in our study.

Softening during fruit ripening is a change in fruit texture that is determined by cell wall metabolic activity^15^. Pectin is the main component of the cell wall, and the enzymes responsible for the metabolism of pectin during fruit ripening and softening include PME, PG and PL^15^. In our study, seven key genes in the pectin metabolism pathway of apricot fruit ripening and softening were enriched. Among these genes, members of the PaPG gene family play important roles in pectin hydrolysis in apricot fruit. We identified three PaPG genes. There were significant differences in the expression levels of the three genes from L63D to L77D, and fruit firmness also decreased significantly from L63D to L77D. PaPME family genes are mainly involved in pectin demethylation and depolymerization. PaPL family genes are mainly involved in pectin lysis (particularly PARG16026). The three gene families function together to cause fruit ripening and softening. The functions of the seven key genes found to be involved in the pectinase metabolism pathway are consistent with those of pectin metabolism genes previously studied in other fruits^16,19,55^.

## Conclusion

In this study, the Illumina sequencing platform was used to analyze the transcriptomes of apricot fruits from the hard-core stage to the mature stage. Among the identified DEGs, 10 genes related to ethylene metabolism and signal transduction and 7 genes related to pectinase were identified. The most critical genes for “Luntaibaixing” apricot were ACS2, ACO, EIN2 and ERF, which participate in the ethylene metabolism pathway and the ethylene signal transduction pathway, and PG and PME, which participate in the pectinase metabolism pathway affecting fruit firmness. Our study provides an important theoretical basis for understanding the ethylene regulation of apricot fruit ripening and lays a foundation for further study of the functional significance of these genes in improving fruit quality traits.

## Supplementary Information

Supplementary Information.
