# Supplementary material for: Transcriptome analysis insight into ethylene metabolism and pectinase activity of apricot (Prunus armeniaca L.) development and ripening
Source: Sci Rep. 2021 Jun 30;11:13569. doi: 10.1038/s41598-021-92832-6 (PMC8245559; doi:10.1038/s41598-021-92832-6)
Supplement: Supplementary file 1 — Supplementary Information. [file 41598_2021_92832_MOESM1_ESM.doc]

**Transcriptome analysis insight into ethylene metabolism and pectinase activity**

**of apricot (*Prunus armeniaca* L.) development and ripening**

Min Xu^a^,Weiquan Zhou^a^, Wenjuan Geng^a^, Shirong Zhao^a^,Yan Pan^b^, Guoquan Fan^c^, Shikui Zhang^c^, Yatong Wang^c^, Kang Liao^a,*^

^a^ Research Centre of Characteristic Fruit Tree, College of Horticulture and Forestry, Xinjiang Agricultural University, Urumqi, Xinjiang 830052, China

^b^ Xinjiang Academy Of Agricultural Sciences, Urumqi, Xinjiang 830052, China

^c^ Luntai National Fruit Germplasm Resources Garden of Xinjiang Academy of Agricultural Sciences, Luntai, Xinjiang, 841600, China

*^*^***Corresponding author:** Kang Liao, **Email:** liaokang01@163.com, **Telephone:** +86-13899825018

**Supplementary material**

Table S1.Primer sequences of key genes used for qRT-PCR

| **Gene** | **Forward** | **Reverse** | **Product size** |
| --- | --- | --- | --- |
| PARG13808 | 5’-AGAACTTCCCAATCATCAAC-3’ | 5’-TCAACCTCTCCACTGTGTCC-3’ | 153 |
| PARG15925 | 5’-AGAAGAAGGACAGCAAACAT-3’ | 5’-GCTTGTGTATCAATACTCCG-3’ | 181 |
| PARG27582 | 5’-GATCACGATCATCACAACCT-3’ | 5’-TTTAGAGTCGAAGCGGAGAG-3’ | 154 |
| PARG12928 | 5’-AAGCTTGTTAGAACCTTGTG-3’ | 5’-TAATTGTCTCCCCTTGACGG-3’ | 177 |
| PARG27966 | 5’-CATCTAATTCCCACGTTATG-3’ | 5’-GGGAGGTCTGTTTTATCTTT-3’ | 163 |
| PARG28436 | 5’-TGGCCAAAGCTCAGATAGAT-3’ | 5’-GTCCCACAAGTTTTGATGAT-3’ | 148 |
| PARG27229 | 5’-TGGACGCTTACGAGCCTATT-3’ | 5’-ATCTCTGGGAGAAGGGAGAC-3’ | 164 |
| PARG17170 | 5’-AGAGGGTGCTTGAGGAGCTT-3’ | 5’-CGTTCTGGAAAAGATTTCCG-3’ | 153 |
| PARG03630 | 5’-AGAGGAAGAGATTTATGGGT-3’ | 5’-GTGTGTGATTCGGTGGAAAG-3’ | 157 |
| PaActin | 5’-CATTCTTCGTCTGGACCTTGC-3’ | 5’-TTGTAGGTAGTCTCATGAATTCC-3’ | 175 |

Table S2.Statistics on the quality and output of the raw data

| Sample | Raw reads | Raw bases | Error rate(%) | Q20(%) | Q30(%) | GC content(%) |
| --- | --- | --- | --- | --- | --- | --- |
| L_42D_1 | 44706624 | 6750700224 | 0.026 | 97.56 | 93.33 | 46 |
| L_42D_2 | 43752844 | 6606679444 | 0.0258 | 97.61 | 93.45 | 46.08 |
| L_42D_3 | 50742968 | 7662188168 | 0.026 | 97.52 | 93.26 | 46.24 |
| L_63D_1 | 46321782 | 6994589082 | 0.0262 | 97.43 | 93.12 | 46.17 |
| L_63D_2 | 48690606 | 7352281506 | 0.0261 | 97.5 | 93.22 | 46.23 |
| L_63D_3 | 52455704 | 7920811304 | 0.026 | 97.58 | 93.3 | 46.4 |
| L_77D_1 | 51116140 | 7718537140 | 0.0262 | 97.52 | 93.1 | 46.33 |
| L_77D_2 | 53454770 | 8071670270 | 0.0262 | 97.52 | 93.05 | 46.41 |
| L_77D_3 | 70204702 | 10600910002 | 0.0259 | 97.58 | 93.41 | 46.42 |

Table S3.The key genes of ethylene metabolism pathway and pectinase metabolism in apricot fruit

|  | **ID** | **Genes** | **Function annotation** | **Gene_name** | **Chrom** | **Start** | **End** |
| --- | --- | --- | --- | --- | --- | --- | --- |
| Ethylene  metabolism | PARG28436 | Pa SAMS | adenosylmethionine decarboxylase activity | Tig00000531_28436 | Tig00000531 | 148057 | 149130 |
|  | PARG18370 | Pa ACS | 1-aminocyclopropane-1-carboxylate synthase 1 | LG5_18370 | LG5 | 18003283 | 18006121 |
|  | PARG27229 | Pa ACS | 1-aminocyclopropane-1-carboxylate synthase | LG8_27229 | LG8 | 15684918 | 15687872 |
|  | PARG13808 | Pa ACO | 1-aminocyclopropane-1-carboxylate oxidase | LG4_13808 | LG4 | 6781276 | 6807870 |
| Signal transduction | PARG19854 | Pa ERF1 | Ethylene response  sensor 1 | LG6_19854 | LG6 | 194107 | 205508 |
|  | PARG08711 | Pa CTR1 | Ethylene-responsive protein kinase | LG2_08711 | LG2 | 38645440 | 38654706 |
|  | PARG02216 | Pa EIN2 | Ethylene-insensitive protein 2 | LG1_02216 | LG1 | 17648356 | 17657828 |
|  | PARG03630 | Pa ETR2 | Ethylene receptor 2 | LG2_03630 | LG2 | 2329824 | 2334428 |
|  | PARG17170 | Pa EIL1 | Ethylene insensitive 3-like 1 protein | LG5_17170 | LG5 | 8482766 | 8486578 |
|  | PARG03138 | Pa ERF | Ethylene-responsive transcription factor 1B-like | LG1_03138 | LG1 | 23245601 | 23246359 |
| Pectinase  metabolism | PARG10145 | Pa PG | Polygalacturonase | LG3_10145 | LG3 | 7933350 | 7936161 |
|  | PARG20309 | Pa PL/PG | Pectate lyase 3 | LG6_20309 | LG6 | 2829136 | 2832617 |
|  | PARG12928 | Pa PL/PG | Pectate lyase superfamily protein/polygalacturonase | LG4_12928 | LG4 | 1473766 | 1476973 |
|  | PARG27582 | Pa PME | Pectinesterase 63 | LG8_27582 | LG8 | 17639871 | 17642291 |
|  | PARG19839 | Pa PME | Pectinesterase | LG6_19839 | LG6 | 136603 | 139094 |
|  | PARG16026 | Pa PL | Pectate lyase | LG4_16026 | LG4 | 24253631 | 24255704 |
|  | PARG18623 | Pa PL | pectate lyase activity | LG5_18623 | LG5 | 19757810 | 19764282 |
